# Association between sleep duration and disability in activities of daily living among Chinese older adults: a nationwide observational study

**DOI:** 10.3389/fpubh.2025.1580101

**Published:** 2025-05-21

**Authors:** Dahuan Cai, Yanxin Zeng, Min Chen, Yun Zhong, Yilin Quan, Mengliang Ye, Xiaoxiao Huang

**Affiliations:** ^1^College of Public Health, Chongqing Medical University, Chongqing, China; ^2^Nanan District Center for Disease Control and Prevention, Chongqing, China; ^3^College of Exercise Medicine, Chongqing Medical University, Chongqing, China

**Keywords:** sleep duration, activity of daily living, physical activity, older adults, observational study, disability, CLHLS

## Abstract

**Background:**

This study aims to explore the relationship between sleep duration and Activity of Daily Living (ADL) disability among older adults in China. ADL disability severely impacts the quality of life of older adults and is associated with various physical and mental health issues. With the aging population in China, the burden of ADL disability is increasing.

**Methods:**

Data were sourced from the 2018 national follow-up of the Chinese Longitudinal Healthy Longevity Survey (CLHLS), including 9,572 participants aged 65 and above. Sleep duration was assessed via self-reported questionnaire and categorized into short (<7 h), medium (7–8 h), and long (≥9 h). ADL disability was evaluated through Basic Activities of Daily Living (BADL) and Instrumental Activities of Daily Living (IADL). Logistic regression models were used to analyze the relationship between sleep duration and ADL disability, with subgroup analyses conducted to explore differences by gender and physical activity.

**Results:**

The study found a significant non-linear relationship between sleep duration and ADL disability. Compared to older adults with a sleep duration of 7–8 h, those with over 9 h of sleep had a significantly higher risk of BADL and IADL disability (OR = 1.36, OR = 1.35). Subgroup analyses indicated that this relationship existed among older adults of different genders, age, and physical activity levels.

**Conclusion:**

For older adults in China, maintaining a sleep duration of 7–8 h may be an effective strategy for preventing ADL disability. Both excessively long and short sleep duration are associated with an increased risk of ADL disability in this population.

## 1 Introduction

Activities of Daily Living (ADL) refer to the basic competencies required for individuals to independently care for themselves, including dressing, eating, and bathing (1). ADL disability is a significant health issue that can lead to decreased quality of life and heightened vulnerability to the surrounding environment (2). Presently, ADL disability is primarily evaluated through Basic Activities of Daily Living (BADL) and Instrumental Activities of Daily Living (IADL) (3, 4). Existing research has demonstrated that ADL disability is strongly associated with physical and mental health issues, including depression, cognitive impairment, frailty, and sarcopenia (5–7). Beyond this, demographic factors, lifestyle behaviors, and health conditions are also significantly linked with ADL disability (8–10). ADL disability not only severely affects the individual patient and family but also poses significant challenges to socio-economic development and the public health system (11). Currently, with the aging population, China is facing an increasing burden of ADL disability, which has become a significant public health issue. A study indicates that the prevalence of ADL disability among individuals aged 60 years and older in China is 23.8% (12). By 2026, the number of individuals aged 65 years and older with ADL disabilities is projected to reach 96.2 million (13). Therefore, to mitigate the burden of ADL disability on individuals, families, and society, it is crucial to identify its risk factors and implement corresponding preventive and intervention measures.

Sleep duration is a critical factor influencing the physical and mental health of older adults and is associated with increased risks of cognitive impairment, cardiovascular disease, type 2 diabetes, and sarcopenia, among others (14–17). Numerous studies have demonstrated a statistically significant ‘U-shaped’ or ‘J-shaped’ association between sleep duration and conditions such as cognitive impairment, depression, and various chronic diseases (18–24). This suggests that patients may face an elevated risk of cognitive impairment, depressive symptoms, and various chronic diseases when sleep duration is either too long or too short. Additionally, other studies have reported that patients experience accelerated cognitive decline when sleep duration is either too long or too short (21). Concurrently, it should be noted that differences in sleep duration requirements exist between age groups and genders, which means they are differently affected by sleep duration (25, 26).

Several previous studies have examined the relationship between sleep duration and ADL disability, but findings have been inconsistent. In 2015, the National Sleep Foundation suggested that the recommended amount of sleep for older adults is 7 to 8 h (27). A study based on the Guangxi Longevity Population Database reported that excessive sleep duration (greater than 12 h) is associated with an increased risk of ADL disability in the oldest population (older than 90 years), and that the optimal sleep duration for this population is 8 to 10 h (28). Luo et al. (29) and Zou et al. (30) demonstrated an association between sleep durations of less than 7 h or more than 7 h and an increased risk of ADL disability in individuals aged over 65 years. Meanwhile, Lee et al. (31) found that older adults with greater limitations in basic activities of daily living (BADL) and instrumental activities of daily living (IADL) experienced more difficulty achieving the recommended sleep duration. These inconsistent results may be attributed to differences in study populations, methods, and designs (17). Moreover, socio-economic, cultural, and ethnic factors may contribute to the heterogeneity observed among different countries or regions in this context (32). However, it is worth noting that current research on sleep duration and ADL disability in older adults remains limited, with insufficient exploration of BADL and IADL classifications. In addition, prior studies on the association between sleep duration and ADL disability in older adults have focused primarily on the overall older adult population, without adequately exploring differences in this association by factors such as gender and physical activity. Therefore, further exploration of the relationship between sleep duration and ADL disability in older adults is necessary to enrich the research in this area and provide a basis for future studies.

In summary, this study aimed to further explore the relationship between sleep duration and ADL disability in older adults, based on existing findings and using data from the Chinese Longitudinal Healthy Longevity Survey (CLHLS), to provide a scientific basis for future research and the development of prevention and intervention strategies for ADL disability in older adults.

## 2 Materials and methods

### 2.1 Participants

Data for this study were sourced from the Chinese Longitudinal Healthy Longevity Survey (CLHLS). The CLHLS is a national prospective longitudinal study organized by the Peking University Centre for Healthy Aging and Development (CHADS) to investigate issues related to aging. Since 1998, the CLHLS has conducted eight national surveys. More information can be found in other related topics (33).

**Figure 1:**
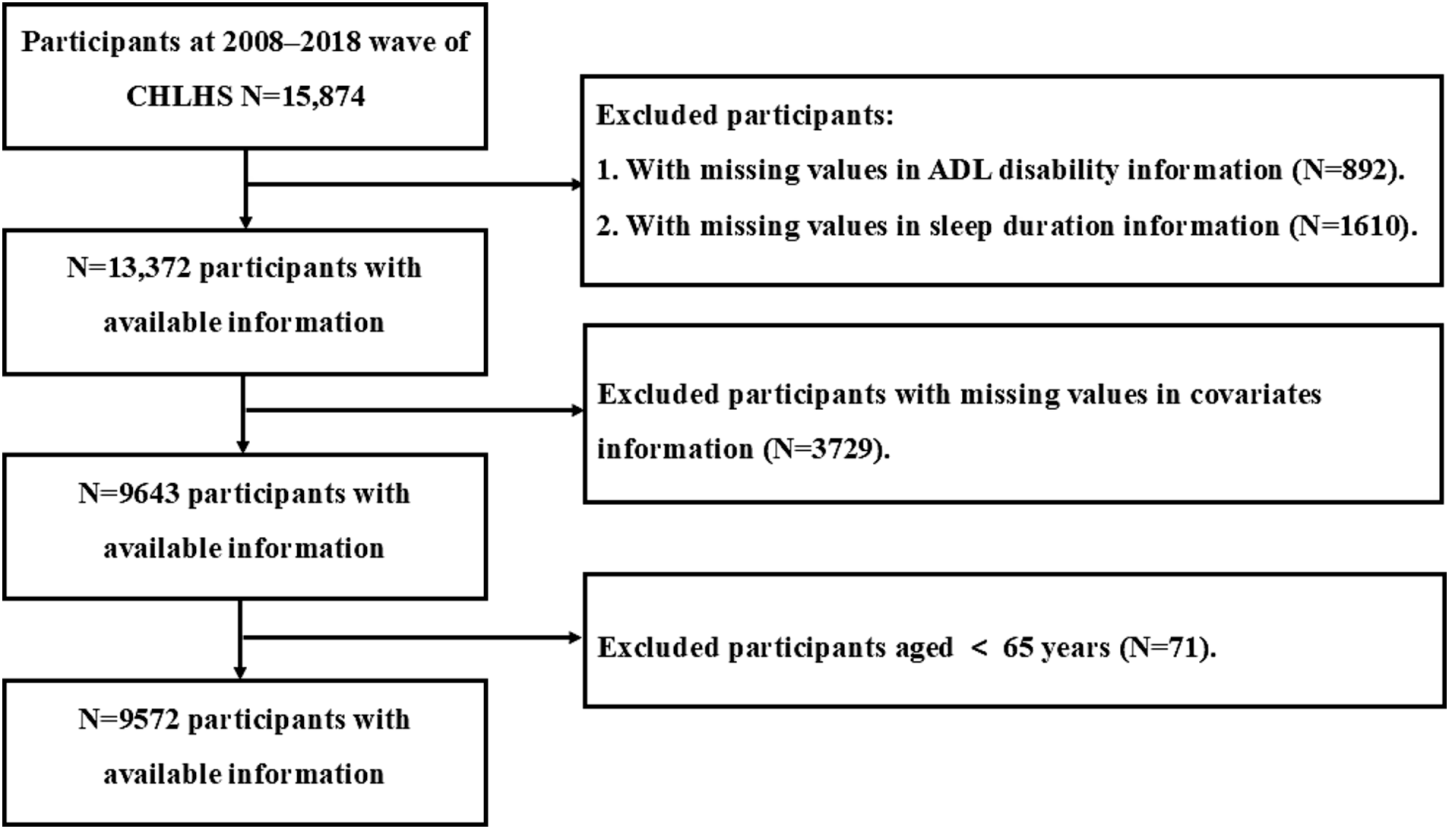
Flowchart of participant screening flowchart.

National follow-up data from CLHLS 2018 were used in this study. To begin with, this study excluded participants with missing data on ADL disability (*n* = 892) and sleep duration (*n* = 1,610); in addition, participants with missing data on covariates were excluded (*n* = 3,729). Finally, participants aged under 65 years were excluded (*n* = 71). Ultimately, 9,572 participants were enrolled in this study. The detailed flowchart of participant inclusion and exclusion for this study is presented in Figure 1.

### 2.2 Assessment of sleep duration

Self-reported sleep duration data were obtained using the question, “How many hours of sleep do you currently get per day?” To better understand the relationship between sleep duration and ADL disability in older adults, this study categorized sleep duration into three groups: short (less than 7 h), medium (7 to 8 h), and long (9 h or more), based on the National Sleep Foundation criteria (27).

### 2.3 Assessment of ADL disability

Participants’ ADL disability status can be assessed through the BADL or IADL. BADL in the CLHLS comprises six items: (1) bathing; (2) eating; (3) dressing; (4) indoor transfers; (5) toileting; (6) incontinence control. IADL comprises eight items: (1) Can you go to your neighbor’s house alone? (2) Can you go out shopping alone? (3) Can you cook alone if needed? (4) Can you do laundry alone if needed? (5) Can you walk 2 miles continuously? (6) Can you lift about 10 pounds (5 kg)? (7) Can you squat down and stand up three times in a row? (8) Can you travel alone on public transport? Participants were considered to have ADL disability if they were unable to perform one or more of the 14 items independently.

### 2.4 Covariates

In order to obtain more reliable results, we included the following covariates that may affect activities of daily living disability based on available references: age (65 to 74, 75 to 89, 90 years or older), gender (male, female), residence (city, town, rural), currently smoking (yes, no), currently drinking (yes, no), marital status (married, unmarried/separated/widowed), physical exercise (yes, no), BMI (obese, overweight, normal, underweight), depression (yes, no), and cognitive impairment (yes, no) (34, 35). Assessment criteria for depression and cognitive impairment are detailed in Supplementary Tables S1, S2.

### 2.5 Ethical considerations

Data for this study were sourced from the CLHLS and used in accordance with the Declaration of Helsinki. Ethical approval was obtained from the Biomedical Ethics Committee of Peking University (IRB00001052-13074) (36). Participants and their families have signed an informed consent form.

### 2.6 Statistical analysis

Statistical analyses of the study were performed using Stata version 18.0 and R version 4.4.1. GraphPad Prism version 9.0 was used to visualize the results of the subgroup analyses. Descriptive statistics were used to summarize the baseline characteristics of the study population. Logistic models were used to estimate the risk ratio (OR) and its corresponding 95% confidence interval (CI) for the association between sleep duration and disability in activities of daily living in older adults. Restricted cubic spline bars were plotted to explore potential non-linear relationships between sleep duration and ADL disability. The Akaike Information Criterion (AIC) was applied to determine the optimal number of knots for the spline. Wald’s test assessed the linearity of the observed relationship. Model 1 was a crude model. Model 2 is adjusted for age, gender, residence, smoking, drinking, marital status, physical exercise, and BMI. Model 3 further adjusted for depression and cognitive impairment based on Model 2. Subgroup analyses based on Model 3 were conducted to examine potential differences in the correlation between sleep duration and ADL disability across gender, age, and physical exercise groups. Statistical significance was set at *p* < 0.05 for two-sided tests.

## 3 Results

### 3.1 Baseline characteristics

This study included 9,572 participants, with 4,575 males (47.8%) and 4,997 females (52.2%). The majority of older adult participants resided in rural areas (43.7%). Among the participants, the largest group (38.3%) reported sleeping 7 to 8 h per day, followed by those who slept less than 7 h (37.8%). In contrast, the smallest proportion (23.9%) reported sleeping more than 9 h. The survey results showed that 1,482 participants (15.5%) experienced BADL disability, and 5,552 participants (58.0%) had IADL disability. The 2018 CLHLS data showed that ADL had good internal consistency, with a Cronbach’s alpha of 0.82.

In this study, significant differences in BADL and IADL were found between groups of older adult participants with different sleep durations, ages, genders, smoking, drinking, marital status, physical exercise, BMI, depression, and cognitive impairment (*p* < 0.05). All these associations were statistically significant at the *p* < 0.05 level, as detailed in Table 1.

**Table 1:** Baseline characteristics of participants.

Variables	BADL disability		*χ* ^2^	*p*-value	IADL disability		*χ* ^2^	*P*-value
	Yes	No			Yes	No		
Sleep duration, *n* (%)								
<7 h	533 (36.0)	3,087 (38.2)	95.47	<0.001	2,165 (39.0)	1,455 (36.2)	157.75	<0.001
7–8 h	453 (30.5)	3,209 (39.6)			1857 (33.4)	1805 (44.9)		
≥9 h	496 (33.5)	1794 (22.2)			1,530 (27.6)	760 (18.9)		
Age, *n* (%)								
65–74	77 (5.2)	2,511 (31.0)	1359.05	<0.001	590 (10.6)	1998 (49.7)	2514.16	<0.001
75–89	388 (26.2)	3,793 (46.9)			2,432 (43.8)	1749 (43.5)		
≥90	1,017 (68.6)	1786 (22.1)			2,530 (45.6)	273 (6.8)		
Gender, *n* (%)								
Male	584 (39.4)	3,991 (49.3)	49.46	<0.001	2,219 (40.0)	2,356 (58.6)	324.67	<0.001
Female	898 (60.6)	4,099 (50.7)			3,333 (60.0)	1,664 (41.4)		
Residence, *n* (%)								
City	509 (34.3)	1774 (21.9)	111.88	<0.001	1,310 (23.6)	973 (24.2)	3.08	0.214
Town	451 (30.4)	2,658 (32.9)			1843 (33.2)	1,266 (31.5)		
Rural	522 (35.2)	3,658 (45.2)			2,399 (43.2)	1781 (44.3)		
Currently smoking, *n* (%)								
Yes	160 (10.8)	1,465 (18.1)	47.52	<0.001	687 (12.4)	938 (23.3)	198.70	<0.001
No	1,322 (89.2)	6,625 (81.9)			4,865 (87.6)	3,082 (76.7)		
Currently drinking, *n* (%)								
Yes	134 (9.0)	1,418 (17.5)	66.40	<0.001	644 (11.6)	908 (22.6)	207.22	<0.001
No	1,348 (91.0)	6,672 (82.5)			4,908 (88.4)	3,112 (77.4)		
Marital status, *n* (%)								
Married	328 (22.1)	4,190 (51.8)	442.14	<0.001	3,730 (67.2)	1,324 (32.9)	1097.39	<0.001
Unmarried/separated/widowed	1,154 (77.9)	3,900 (48.2)			1822 (32.8)	2,696 (67.1)		
Physical exercise, *n* (%)								
Yes	305 (20.6)	3,160 (39.1)	185.22	<0.001	1,540 (27.7)	1925 (47.9)	409.83	<0.001
No	1,177 (79.4)	4,930 (60.9)			4,012 (72.3)	2095 (52.1)		
Body mass index, *n* (%)								
Underweight	318 (21.5)	1,035 (12.8)	89.06	<0.001	998 (18.0)	355 (8.9)	205.55	<0.001
Normal	871 (58.8)	4,924 (61.0)			3,330 (60.0)	2,465 (61.4)		
Overweight	242 (16.3)	1809 (22.4)			1,007 (18.2)	1,044 (26.0)		
Obese	51 (3.4)	309 (3.8)			212 (3.8)	148 (3.7)		
Depression, *n* (%)								
Yes	292 (19.7)	1,002 (12.4)	57.37	<0.001	975 (17.6)	319 (7.9)	184.80	<0.001
No	1,190 (80.3)	7,088 (87.6)			4,577 (82.4)	3,701 (92.1)		
Cognitive impairment, *n* (%)								
Yes	408 (27.5)	390 (4.8)	845.31	<0.001	780 (14.0)	18 (0.4)	564.46	<0.001
No	1,074 (72.5)	7,700 (95.2)			4,772 (86.0)	4,002 (99.6)		

BADL refers to basic activities of daily living. IADL refers to instrumental activities of daily living.

### 3.2 Association of sleep duration with disability in activities of daily living

**Figure 2:**
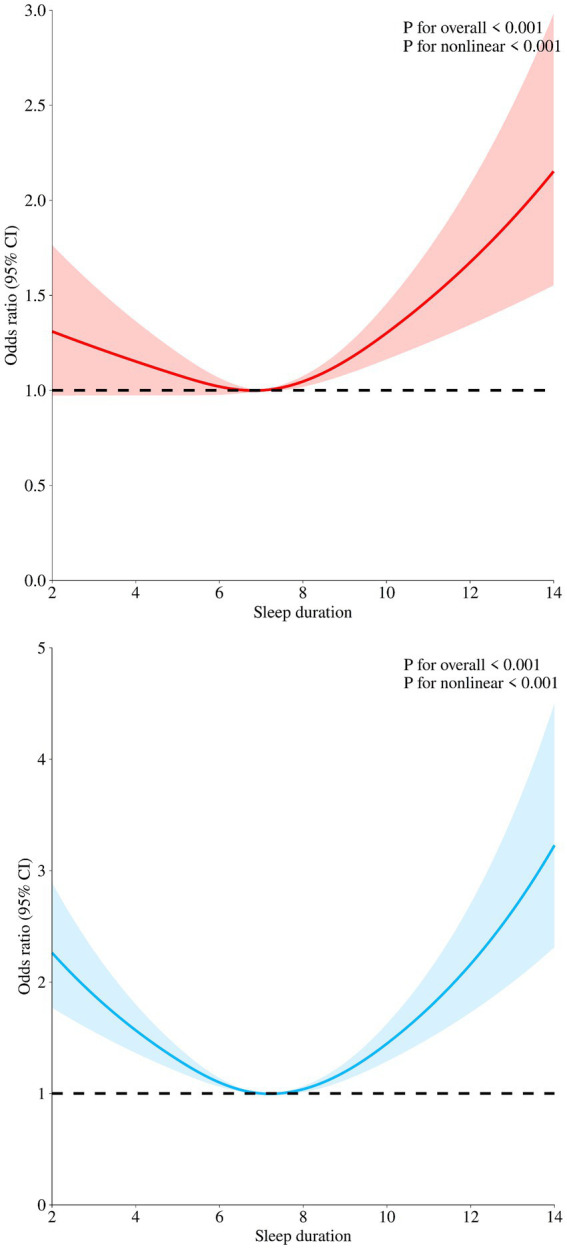
Restricted cubic spline regression analysis for sleep duration and BADL and IADL. The red curve indicates the association between sleep duration and BADL in older adults. The blue curve indicates the association between sleep duration and IADL in older adults.

Figure 2 demonstrates a significant statistical association between sleep duration and both Basic Activities of Daily Living (BADL) and Instrumental Activities of Daily Living (IADL) (*p* < 0.001). A non-linear relationship is observed between sleep duration and BADL or IADL (*p* < 0.001). In Model 1 (shown in Table 2), compared to individuals with 7–8 h of sleep, those with less than 7 h and more than 9 h of sleep have a significantly higher risk of BADL disability (OR = 1.22, *p* = 0.003; OR = 1.96, *p* < 0.001). In Model 3, after adjusting for all covariates, compared to those with 7–8 h of sleep, the risk of BADL disability in individuals with less than 7 h of sleep is no longer statistically significant (*p* = 0.421), while those with more than 9 h of sleep still exhibit a higher risk of BADL disability (OR = 1.36, *p* < 0.001). Similarly, in Model 1 (Supplementary Table S4), individuals with less than 7 h and more than 9 h of sleep have a significantly higher risk of IADL disability (OR = 1.45, *p* < 0.001; OR = 1.96, *p* < 0.001). In Model 3, the risk of IADL disability remains significantly higher in those with less than 7 h and more than 9 h of sleep (OR = 1.17, *p* = 0.007; OR = 1.35, *p* < 0.001).

**Table 2:** Association of sleep duration with the incidence of BADL disability risk.

Variables	Sleep duration	Model 1		Model 2		Model 3	
		OR (95%CI)	*P*-value	OR (95%CI)	*P*-value	OR (95%CI)	*P*-value
Sleep duration	7–8 h	Reference		Reference		Reference	
	<7 h	1.22 (1.07–1.40)	0.003	1.12 (0.96–1.30)	0.143	1.07 (0.91–1.24)	0.421
	≥9 h	1.96 (1.70–2.25)	<0.001	1.40 (1.20–1.63)	<0.001	1.36 (1.16–1.60)	<0.001
Age	65–74			Reference		Reference	
	75–89			2.80 (2.17–3.62)	<0.001	2.68 (2.07–3.47)	<0.001
	≥90			12.31 (9.47–16.01)	<0.001	10.16 (7.79–13.27)	<0.001
Gender	Male			Reference		Reference	
	Female			1.07 (0.93–1.23)	0.346	1.00 (0.87–1.15)	0.997
Residence	City			Reference		Reference	
	Town			0.46 (0.39–0.54)	<0.001	0.43 (0.36–0.51)	<0.001
	Rural			0.39 (0.33–0.45)	<0.00	0.36 (0.31–0.42)	<0.001
Currently smoking	Yes			Reference		Reference	
	No			1.05 (0.86–1.29)	0.640	1.02 (0.83–1.25)	0.845
Currently drinking	Yes			Reference		Reference	
	No			1.55 (1.26–1.91)	<0.001	1.15 (1.22–1.87)	<0.001
Marital status	Married			Reference		Reference	
	Unmarried/separated/widowed			1.46 (1.25–1.71)	<0.001	1.38 (1.18–1.63)	<0.001
Physical exercise	Yes			Reference		Reference	
	No			2.08 (1.79–2.42)	<0.001	1.88 (1.62–2.19)	<0.001
Body mass index	Normal			Reference		Reference	
	Underweight			1.16 (0.99–1.36)	0.075	1.09 (0.93–1.29)	0.287
	Overweight			1.11 (0.94–1.32)	0.225	1.15 (0.97–1.37)	0.115
	Obese			1.24 (0.88–1.74)	0.213	1.24 (0.88–1.75)	0.228
Depression	Yes			Reference		Reference	
	No					0.69 (0.58–0.81)	<0.001
Cognitive impairment	Yes			Reference		Reference	
	No					0.31 (0.26–0.37)	<0.001

Model 1 was a crude model. Model 2 is adjusted for age, gender, residence, smoking, drinking, marital status, physical exercise, and BMI. Model 3 further adjusted for depression and cognitive impairment based on Model 2.

### 3.3 Subgroup analysis of sleep duration and disability in activities of daily living

**Figure 3:**
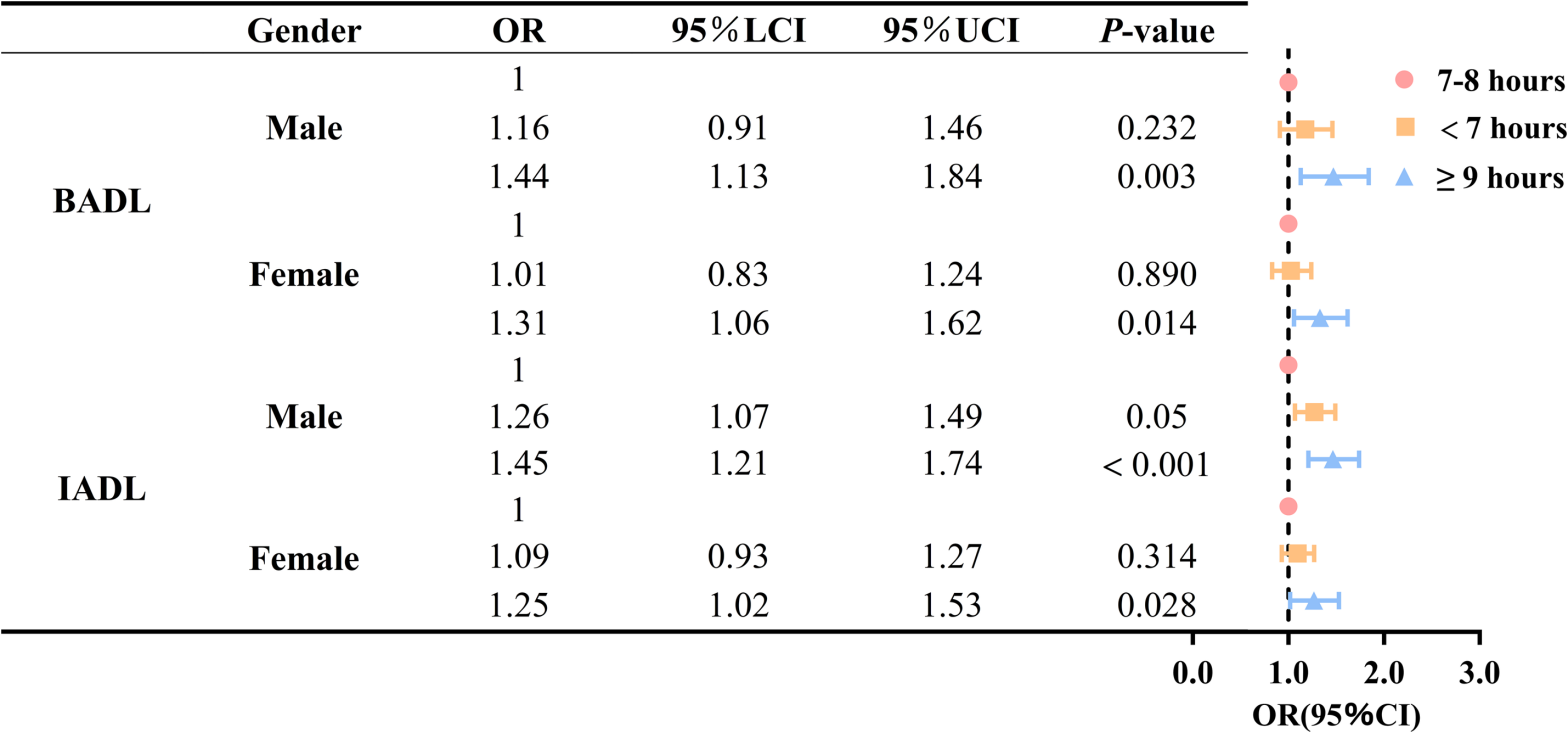
Association between sleep duration and disability in activities of daily living based on gender stratification.

**Figure 4:**
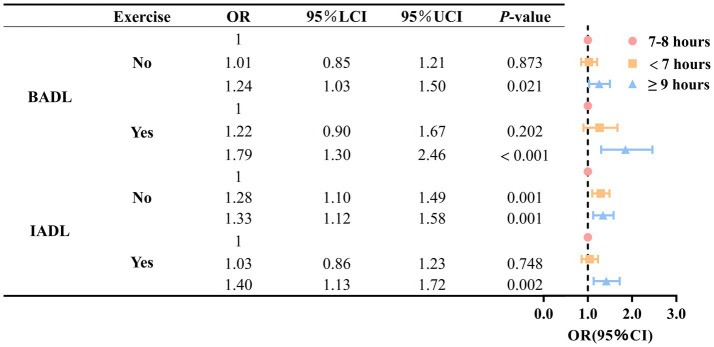
Association between sleep duration and disability in activities of daily living based on physical activity stratification.

To further explore the association between sleep duration and ADL disability in older adult individuals with different gender, physical exercise, and age levels, we conducted subgroup analyses based on gender, physical exercise, and age (as shown in Figures 3, 4 and Supplementary Figure S1). The results indicated that, compared to older adult individuals with 7–8 h of sleep, those with sleep durations of 9 h or more had a higher risk of IADL and BADL disability, regardless of whether they were male or female participants. Among older adult individuals who engage in physical exercise, no significant difference in the risk of BADL and IADL disability was observed between those who slept less than 7 h and those who slept 7–8 h (*p* = 0.202, *p* = 0.748).

## 4 Discussion

This study examined the association between sleep duration and ADL disability in older adults. The results of the study demonstrated a non-linear relationship between sleep duration and BADL and IADL disability in older adults. For older adults, 7–8 h of sleep is optimal, and either too long or too short a sleep duration is associated with an increased risk of ADL disability. Previous studies have demonstrated that BADL and IADL disabilities are significant risk factors for older adults who struggle to meet the recommended sleep duration (31). Thus, a bidirectional relationship may exist between sleep duration and ADL disability in older adults. Additionally, the association between sleep duration and ADL disability varied by gender and physical exercise. Specifically, when sleep duration is 9 h or more, older men exhibited a greater proportion of the increased risk of BADL disability compared to older women. When sleep duration was either too long or too short, the proportion of ADL disability risk increased more among older adults who engaged in physical exercise than among those who did not.

Our study demonstrated a U-shaped relationship between sleep duration and ADL disability in older adults, with both shorter and longer sleep durations than optimal being associated with an increased risk of ADL disability. Multiple physical and psychological risk factors may mediate this association, including depressive symptoms, cognitive impairment, and chronic diseases like cardiovascular disease and diabetes. Depressive symptoms are more common in older adults (37, 38). Yan et al. reported that ADL disability is strongly associated with depressive symptoms in older adults (39, 40). Older adults with depressive symptoms are more likely to experience physical dysfunction and ADL disability, which may in turn contribute to the development of depressive symptoms (40). Concurrently, sleep duration also demonstrates a bidirectional relationship with depressive symptoms (41). In this context, an optimal sleep duration of 8 h reduces the risk of depression, whereas both prolonged and shortened sleep durations increase this risk (42).

Cognitive impairment also plays an important role in this association. Several previous studies have demonstrated that cognitive decline directly impacts ADL ability in older adults, with this effect being particularly pronounced in those with dementia and cognitive loss (43, 44). Additionally, through a comprehensive analysis of two nationally representative aging cohorts, Ma et al. indicated a U-shaped relationship between sleep duration and cognitive functioning (45). Both shorter (6 h or less) and longer (8 h or more) sleep durations negatively affect cognitive performance in older adults, increasing the risk of cognitive impairment and dementia, particularly with extreme sleep durations of 4 h or less or 10 h or more per night (45, 46).

Furthermore, the role of various chronic diseases in the relationship between sleep duration and ADL disability in older adults is noteworthy. Existing studies indicate that chronic degenerative diseases (CDDs), including obesity, cardiovascular disease, diabetes, and inflammation, are among the primary causes of ADL disability in older adults (8, 47–49). A Polish study identified the comorbidity of chronic diseases as the primary risk factor for BADL disability in older adults, with the risk of BADL disability increasing as the number of coexisting chronic diseases rises (50). The pain caused by these chronic diseases may also limit physical activity, thus inducing IADL disability in older adults (50). Simultaneously, an association between sleep duration and chronic disease has been demonstrated. Dashti et al. (51) investigated the relationship between short sleep duration and chronic diseases, concluding that short sleep duration is associated with increased total calorie, fat, and protein intake, thereby contributing to chronic diseases such as obesity, type 2 diabetes, and cardiovascular disease. Simultaneously, the risk of certain chronic diseases also demonstrates a U-shaped relationship with sleep duration, where only an appropriate sleep duration can maintain a low risk, as seen in chronic kidney disease and various cardiovascular diseases (52–54). This appropriate sleep duration tends to be closer to the recommended time, between 6 and 9 h.

Subgroup analyses demonstrate the robustness of the study findings. Research shows that both long and short sleep times are associated with increased ADL disability risk in the older adult, regardless of gender, physical status, or age subgroup. Moreover, subgroup analyses revealed gender and physical exercise differences in the association between sleep duration and ADL disability in older adults. Firstly, when sleep duration is 9 h or more, older men exhibited a greater proportion of the increased risk of ADL disability compared to older women. This may be attributed to the physiological differences between men and women. Research indicates that women require more sleep than men (55, 56). Furthermore, as women are required to perform a significant portion of unpaid work (e.g., care for family members) in addition to paid work, they are more likely to experience sleep disorders and have lower sleep quality compared to men (55, 57). Therefore, appropriately extending sleep duration may improve their physical health while mitigating the adverse effects of prolonged sleep.

Secondly, when sleep duration was too long or too short, the increase in risk of ADL disability was more pronounced in older adults who were physically exercising compared to those who were not. This finding differs from previous studies that typically concluded older adults participating in physical exercise have a relatively low risk of ADL disability (58–61). This may be attributed to the fact that the CLHLS questionnaire, when assessing participants’ physical exercise, only indicates whether they are currently exercising, without evaluating the frequency or intensity of their physical exercise. Therefore, this subgroup analysis can only reflect the magnitude of the change in the risk of ADL disability in older adults caused by excessive or insufficient sleep duration, but not the intensity of the original risk or the intensity of the increase. The conclusion should thus be interpreted with caution. It may also be that older adults who participate in physical activity are in better physical condition, sleep better, and require less sleep than those who do not participate in physical activity. Therefore, sleeping too much may have a more pronounced effect on their increased risk of disability in daily mobility.

It is also worth noting that the distribution of sleep duration among Chinese older adults is distinct from other global regions. Our study found that the proportion of Chinese seniors with optimal sleep duration is lower than that in other global regions, while the proportion of those with short sleep duration is higher (62–65). This may be attributed to a range of factors including culture, lifestyle, social support systems, health behaviors, and psychological factors (66, 67). Simultaneously, this may also indicate that sleep duration has a greater influence on the risk of ADL disability in Chinese older adults than in those from other countries and regions. Based on these findings, future studies should focus more on Chinese older adults with short sleep duration.

Finally, current evidence indicates that patients’ sleep duration can be improved through pharmacotherapy, including benzodiazepines, antihistamines, etc. (68). Nevertheless, certain medications may heighten the risk of adverse effects in older adults, potentially compromising their ADL capacity (69). For instance, benzodiazepines may impair cognitive performance and neuromotor function in older adults (69, 70). Therefore, pharmacological interventions targeting adverse sleep duration in older adults must be exercised with caution. In addition, studies have shown that certain lifestyle habits related to health are associated with sleep duration, such as frequent alcohol use, heavy coffee intake, and bedtime mobile phone use (71). Thus, clinicians should prioritize non-pharmacological interventions in clinical practice and aim to improve patients’ sleep duration through behavioral or psychological interventions wherever feasible. Concurrently, policymakers should strive to promote healthy lifestyle habits and enhance public awareness of sleep hygiene through community-based health education.

## 5 Innovations and limitations

This study has several notable strengths. To begin with, the data used in this study are nationally representative; In addition, the study is clinically significant for maintaining the physical and mental health and self-care capacity of Chinese older adults. However, this study also has several limitations. First, the data used in this study were derived from a self-assessment questionnaire, which may introduce recall bias. Second, the cross-sectional design limits the exploration of causal relationships. Finally, the CLHLS database records only whether participants currently engage in physical exercise, without assessing the frequency or intensity of physical exercise, which may potentially confound the study results.

## 6 Conclusion

In conclusion, a sleep duration of 7–8 h seems to be optimal for Chinese older adults, and too long or too short a sleep duration is associated with an increased risk of ADL disability in this population. Maintaining a sleep duration of 7–8 h may be an effective strategy for preventing ADL disability in Chinese older adults.

## Data Availability

The datasets presented in this study can be found in online repositories. The names of the repository/repositories and accession number (s) can be found: https://opendata.pku.edu.cn/dataverse/CHADS.
