# Supplementary material for: Association between sleep duration and disability in activities of daily living among Chinese older adults: a nationwide observational study
Source: Front Public Health. 2025 May 21;13:1580101. doi: 10.3389/fpubh.2025.1580101 (PMC12133472; doi:10.3389/fpubh.2025.1580101)
Supplement: Supplementary file 1 [file Data_Sheet_1.PDF]

Supplementary materials

Contents

1. Figure S1 Association between sleep duration and disability in activities of daily living based on age stratification..01

2. Table S1 The mini-mental state examination (MMSE) of the Chinese version in the CLHLS..02

3. Table S2 Depression (CES-D) Scale in the CLHLS..03

4. Table S3. Assessment of Activities of Daily Living (ADL) in the CLHLS..........05

5. Table S4 Association of sleep duration with the incidence of IADL disability risk...06

6. Table S5 Association of sleep duration with ADL entries in older adults.......... 08

7. Table S6 Relationship between sleep duration and incidence of ADL disability risk (raw data)..10

	Age	OR	95%LCI	95%UCI	*P*-value
		1			
	**65-74**	0.85	0.49	1.47	0.557
		1.55	0.85	2.80	0.150
		1			
**BADL**	**75-89**	1.12	0.87	1.45	0.380
		1.44	1.07	1.92	0.015
		1			
	**≥ 90**	1.08	0.88	1.32	0.485
		1.32	1.07	1.62	0.008
		1			
	**65-74**	1.22	0.98	1.51	0.071
		1.26	0.96	1.66	0.101
		1			
**IADL**	**75-89**	1.12	0.97	1.30	0.125
		1.39	1.16	1.65	< 0.001
		1			
	**≥ 90**	1.36	0.98	1.87	0.063
		1.40	1.02	1.93	0.038

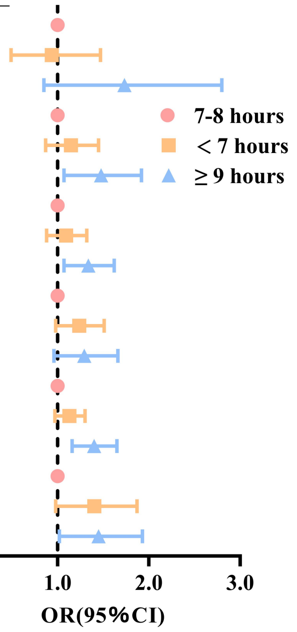

Figure S1 Association between sleep duration and disability in activities of daily living based on age stratification.

Table S1 The mini-mental state examination (MMSE) of the Chinese version of the CLHLS

Domains	Questions	Scores
Orientation	1. What time of day is it right now (morning, afternoon, evening)?	1
	2. What is the month (Western or Chinese calendar) right now?	1
	3. What is the date (Chinese calendar day and month) of the mid-autumn festival?	1
	4. What is the season right now, spring, summer, fall, winter?	1
	5. What is the name of this district or town?	1
	6. Please name as many kinds of food as possible in 1 minute.	7
Registration	1. I am now going to test your memory. I will mention three objects. Please repeat these three objects (table, apple, clothes).	3
Attention and calculation	1. I will ask you to spend 3 dollars from 20 dollars, then you must spend 3 dollars from the number you arrived at and continue to spend 3 dollars until you are asked to stop.	5
	2. Ask the interviewee to draw the figure on B Card	1
Recall	1. Please repeat the three words (in any order) that I asked you to repeat a little while ago.	3
Language	1. Give the interviewee a pen and then a watch and ask what these objects are called(pen, watch).	2
	2. I will now ask you to repeat the following sentence: ‘What you plant, what you will get.’	1
	3. I will give you a piece of paper. You must take the paper using your right hand, fold it in the middle using both hands, and place the paper on the floor.	3

Note: total score ranging from 0 to 30.
Participants' cognitive function is defined as normal when their cognitive scores are ⩾24, while scores between 0 and 23 indicate cognitive impairment.

Table S2 Depression (CES-D) Scale in the CLHLS

Question	Frequency	Scores
1. Are you bothered by things that don't usually bother you?	always	3
	often	2
	sometimes	1
	seldom	0
	never	
2. Do you have trouble focusing on what you were doing?	always	3
	often	2
	sometimes	1
	seldom	0
	never	
3. Do you feel sad, blue, or depressed?	always	3
	often	2
	sometimes	1
	seldom	0
	never	
4. Do you feel the older you get, the more useless you are, and have trouble doing anything?	always	3
	often	2
	sometimes	1
	seldom	0
	never	
5. Do you feel hopeful about the future?	always	0
	often	
	sometimes	1
	seldom	2
	never	3
6. Do you often feel fearful or anxious?	always	3
	often	2
	sometimes	1
	seldom	0
	never	
7. Are you as happy as when you were younger?	always	0
	often	

	sometimes	1
	seldom	2
	never	3
8. Do you often feel lonely and isolated?	always	3
	often	2
	sometimes	1
	seldom	0
	never	
9. Do you feel you could not get "going"?	always	3
	often	2
	sometimes	1
	seldom	0
	never	
10. How about the quality of your sleep?	very good	0
	good	
	so so	1
	bad	2
	very bad	3

Note: total score ranging from 0 to 30.

Participants scored 0-9 points have no depression, whereas those with a score of ≥10 points are considered to have depression.

Table S3. Assessment of Activities of Daily Living (BADL) in the CLHLS

Domains	Questions	Frequency
BADL	1. Bathing – either sponge bath, tub bath, shower or washing the body.<br>2. Dressing – gets clothes from closets and drawers – including underwear, outer garments, and fasteners (including suspenders, if worn).<br>3. Toilet – going to the toilet; and cleaning oneself afterwards.<br>4. Indoor Transfer<br>5. Continence<br>6. Eating	1. Yes, independently;<br>2. Yes, but need some assistance;<br>3. No, can't.
IADL	7. Can you visit your neighbors by yourself?<br>8. Can you go shopping by yourself?<br>9. Can you cook a meal by yourself whenever necessary?<br>10. Can you wash clothing by yourself whenever necessary?<br>11. Can you walk continuously for 1 kilometer at a time by yourself?<br>12. Can you lift a weight of 5kg, such as a heavy bag of groceries?<br>13. Can you continuously crouch and stand up three times?<br>14. Can you take public transportation by yourself?	

Note: If a participant is unable to perform one or more of the 14 items listed above without assistance, they will be classified as ADL disabled.

Table S4 Association of sleep duration with the incidence of IADL disability risk.

Variables	Sleep duration	Model 1		Model 2		Model 3	
		OR(95%CI)	*P-value*	OR(95%CI)	*P-value*	OR(95%CI)	*P*-value
Sleep duration	7-8hours	Reference		Reference		Reference	
	<7hours	1.45(1.32-1.59)	<0.001	1.27(1.14-1.42)	<0.001	1.17(1.04-1.31)	0.007
	≥9hours	1.96(1.76-2.18)	<0.001	1.38(1.21-1.57)	<0.001	1.35(1.18-1.54)	<0.001
Age	65-74			Reference		Reference	
	75-89			4.29(3.81-4.84)	<0.001		
	≥90			22.45(18.88-26.70)	<0.001		
Gender	Male			Reference		Reference	
	Female			1.66(1.49-1.85)	<0.001		
Residence	City			Reference		Reference	
	Town			0.97(0.85-1.12)	0.694	0.95(0.83-1.09)	0.441
	Rural			0.89(0.78-1.01)	0.076	0.88(0.77-1.00)	0.046
Currently smoking	Yes			Reference		Reference	
	No			1.24(1.07-1.43)	0.003	1.25(1.08-1.44)	0.002
Currently drinking	Yes			Reference		Reference	
	No			1.51(1.32-1.74)	<0.001	1.46(1.27-1.69)	<0.001
Marital status	Married			Reference		Reference	
	Unmarried/separated/widowed			1.51(1.36-1.68)	<0.001	1.45(1.30-1.61)	<0.001
Physical exercise	Yes			Reference		Reference	
	No			2.01(1.81-2.23)	<0.001	1.86(1.67-2.07)	<0.001
Body mass index	Normal			Reference		Reference	
	Underweight			1.20(1.03-1.41)	0.023	1.16(0.98-1.36)	0.078
	Overweight			1.12(0.99-1.27)	0.063	1.15(1.02-1.30)	0.026
	Obese			1.49(1.16-1.92)	0.002	1.49(1.16-1.93)	0.002

Depression	Yes	Reference	Reference	
	No		2.18(1.86-2.56)	<0.001
Cognitive impairment	Yes	Reference	Reference	
	No		9.72(5.99-15.77)	<0.001

Note: Model 1 was a crude model. Model 2 is adjusted for age, gender, residence, smoking, drinking, marital status, physical exercise, and BMI. Model 3 further adjusted for depression and cognitive impairment based on Model 2

Table S5 Association of sleep duration with ADL entries in older adults

Independent variables	Sleep duration	OR(95%CI)	*P*-value
Bathing	7-8 hours	Reference	
	<7hours	1.02(0.87-1.19)	0.080
	>9hours	1.35(1.15-1.59)	<0.001
Dressing	7-8 hours	Reference	
	<7hours	0.96(0.78-1.21)	0.785
	>9hours	1.20(0.95-1.51)	0.133
Toilet	7-8 hours	Reference	
	<7hours	0.98(0.77-1.24)	0.863
	>9hours	1.17(0.91-1.51)	0.210
Indoor Transfer	7-8 hours	Reference	
	<7hours	0.82(0.63-1.06)	0.133
	>9hours	1.08(0.83-1.41)	0.573
Continence	7-8 hours	Reference	
	<7hours	1.16(0.82-1.63)	0.397
	>9hours	1.69(1.20-2.37)	0.003
Eating	7-8 hours	Reference	
	<7hours	0.83(0.59-1.15)	0.262
	>9hours	1.12(0.80-1.58)	0.511
Social Mobility	7-8 hours	Reference	
	<7hours	0.99(0.85-1.15)	0.845
	>9hours	1.26(1.07-1.48)	0.005
Shopping Independence	7-8 hours	Reference	
	<7hours	1.06(0.93-1.21)	0.378
	>9hours	1.44(1.25-1.66)	<0.001
Meal Preparation	7-8 hours	Reference	
	<7hours	1.20(1.05-1.36)	0.006
	>9hours	1.47(1.28-1.69)	<0.001
Laundry Management	7-8 hours	Reference	
	<7hours	1.18(1.04-1.35)	0.010
	>9hours	1.31(1.14-1.51)	<0.001
Ambulation Endurance	7-8 hours	Reference	
	<7hours	1.26(1.13-1.40)	<0.001
	>9hours	1.47(1.30-1.66)	<0.001

	7-8 hours	Reference	
Strength Capacity	<7hours	1.22(1.10-1.36)	<0.001
	>9hours	1.29(1.14-1.46)	<0.001
	7-8 hours	Reference	
Lower Limb Function	<7hours	1.26(1.15-1.40)	<0.001
	>9hours	1.28(1.15-1.44)	<0.001
	7-8 hours	Reference	
Transport Autonomy	<7hours	1.20(1.08-1.34)	0.001
	>9hours	1.48(1.30-1.67)	<0.001

Note: Age, gender, residence, smoking, drinking, marital status, physical activity, BMI, depression, and cognitive impairment were included in the model as control variables.

Table S6 Relationship between sleep duration and incidence of ADL disability risk (raw data).

Variables	Sleep duration	Model 1		Model 2		Model 3	
		OR(95%CI)	*P-value*	OR(95%CI)	*P-value*	OR(95%CI)	*P*-value
BADL	7-8hours	Reference		Reference		Reference	
	<7hours	1.22(1.19-1.33)	<0.001	1.11(0.99-1.22)	0.055	1.09(0.97-1.24)	0.158
	≥9hours	2.05(1.88-2.25)	<0.001	1.44(1.29-1.60)	<0.001	1.31(1.15-1.49)	<0.001
IADL	7-8hours	Reference		Reference		Reference	
	<7hours	1.40(1.30-1.51)	<0.001	1.24(1.13-1.37)	<0.001	1.11(1.00-1.24)	0.050
	≥9hours	2.31(2.11-2.53)	<0.001	1.47(1.31-1.65)	<0.001	1.32(1.16-1.50)	<0.001

Note: Model 1 was a crude model. Model 2 is adjusted for age, gender, residence, smoking, drinking, marital status, physical exercise, and BMI. Model 3 further adjusted for depression and cognitive impairment based on Model 2
